# The usefulness of proximal anchor balloon technique during implantation of a cardiac resynchronization therapy device in a patient with complex coronary venous anatomy: a case report

**DOI:** 10.1186/s12872-022-02484-1

**Published:** 2022-02-05

**Authors:** Ravindra Bhardwaj, Amit Chaurasia, Nipun Mahajan, Harvinder Dod, Kuldeep Arora

**Affiliations:** 1 Department of Cardiology, Tagore Hospital and Heart Care Centre, 91 Mahavir Marg, Jalandhar, Punjab 144008 India; 2 Department of Cardiology, Artemis Hospital, Gurgaon, Haryana India; 3 Heart and Vascular Institute of South Arkansas, El Dorado, AR USA

**Keywords:** Heart failure, Cardiac resynchronization therapy, Anchor balloon, Large coronary sinus, Case report

## Abstract

**Background:**

Cardiac resynchronization therapy (CRT) is an accepted device treatment in stable heart failure (HF) patients. In recent years increased awareness of coronary anatomy and implantation techniques have significantly impacted this evolving therapy.

**Case presentation:**

In this article, we present a case describing the usefulness of the proximal balloon anchoring technique to enable initial coronary sinus (CS) cannulization and left ventricular (LV) lead placement in the tortuous coronary sinus during CRT implantation.

**Conclusions:**

The proximal anchor balloon technique can easily enable coronary sinus cannulization and left ventricular lead placement in patients with complex venous anatomy.

**Supplementary Information:**

The online version contains supplementary material available at 10.1186/s12872-022-02484-1.

## Background

In the process of implanting a cardiac resynchronization therapy device, the coronary venous anatomy can make successful implantation difficult or impossible [1]. This case report describes the use of balloons as anchors to enable coronary sinus (CS) cannulization and left ventricular (LV) lead placement in patients with complex venous anatomy.

## Case presentation

**Fig. 1:**
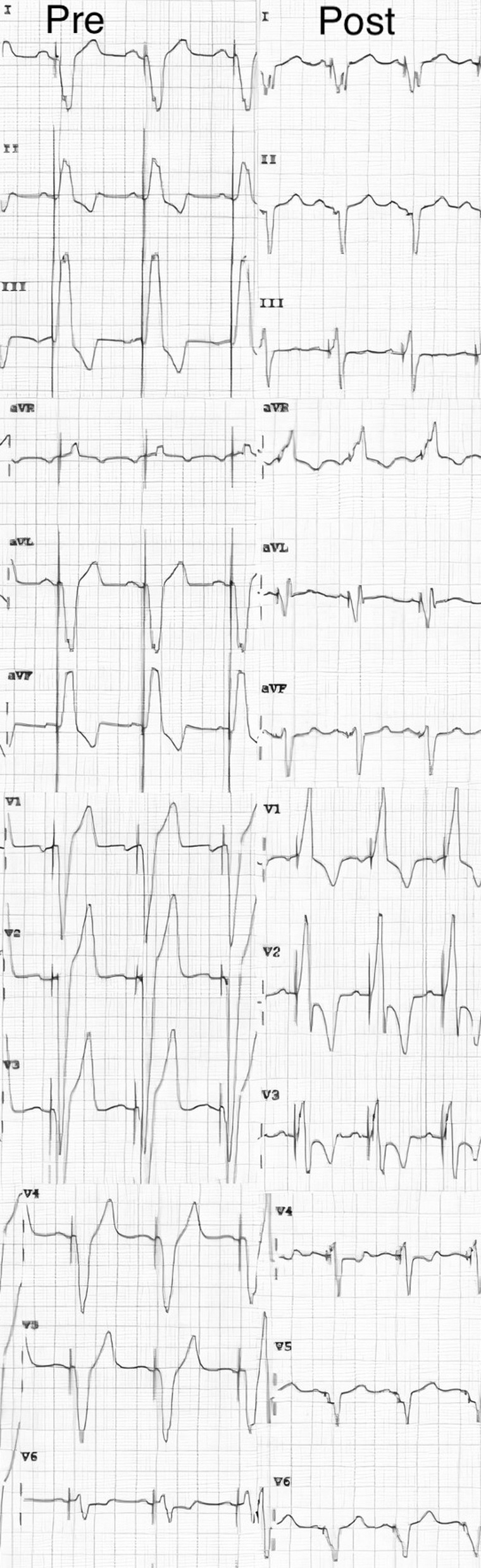
Twelve-lead ECG (25 mm/s) showing sinus rhythm and left bundle branch block with a QRS duration of 160 ms before cardiac resynchronization therapy (Pre) and marked narrowing of the QRS (110 ms) upon implantation of a cardiac resynchronization therapy (Post)

A 70-year-old man presented to the hospital with exertional dyspnea and pedal edema. The patient had a complete heart block one year back, for which he got a DDDR pacemaker implanted. LV function and coronary angiogram were normal at the time of pacemaker implantation. Before presenting to our hospital, the patient was admitted to the local hospital and managed for heart failure.The echocardiography showed a dilated LV with severe LV dysfunction (LV ejection fraction 25%) and plasma B-type natriuretic peptide level was found to be 877 pg/mL. When evaluated at our centre. ECG showed paced rhythm with a QRS interval of 160 ms (Fig. 1 pre). The decrease in ejection fraction was due to RV pacing hence device up-gradation to cardiac resynchronization therapy defibrillator (CRT-D) was planned. An organized timeline of the patient’s symptoms and interventions is presented in Table 1.

**Table 1:** Organized timeline of the patient symptoms and interventions

03/2017	Progressive shortness of breath and angina upon exertion since last few years	New York Heart Association (NYHA) Class III
04/2017	Permanent Pacemaker implantation Heart Failure (HFrEF)	
06/2018	Worsening of shortness of breath and angina upon exertion since last few months	
07/2018	Patient admitted to outside facility	Left ventricular ejection fraction (LVEF) 25%
	Heart Failure (HFrEF)	
	LBBB	ACE inhibitor and beta-blocker
	Reduced left ventricular systolic function	
	Heart Failure medications initiated	
08/2018	Referred to our centre	
	Coronary angiography	No coronary artery disease, slow flow
	Heart failure medication intensified	ACE inhibitor + betablocker + MR antagonist dose increased
08/2018	Heart failure (HFrEF)	NYHA class III
	Left bundle branch block	QRS 160 ms
	Reduced left ventricular systolic function	LVEF 25%
08/2018	CRT D Implantation	QRS 110 ms
09/2018	Heart failure (HFrEF) Patient symptomatically improving	New York Heart Association (NYHA) Class II

A coronary angiogram was done to rule out coronary artery disease (CAD) as a cause for severe LV dysfunction. During the coronary angiogram, levophase was done to visualize the coronary sinus and locate its ostium in the left and right anterior oblique views. After visualizing the anatomy of the CS, the posterolateral vein was pointed out as a possible vessel for LV lead implantation. The posterolateral vein had difficult annatomy as it originated perpendicular to CS. Left subclavian vein puncture was taken, and an LV sheath was inserted. Using AL-1 diagnostic catheter, CS was cannulated through the LV sheath with 0.014 × 180 cm Terumo guidewire. Whisper Extra Support (WES) guidewire was placed in the posterolateral vein. LV lead could not be advanced through the ostium of the posterolateral vein due to difficult angulation. Since the LV lead could not be advanced, a sub-selector catheter was used, but despite this, the LV lead could not be advanced. Hence, a second WES wire was placed in the posterolateral vein. LV wire was inserted into the posterolateral vein with a second support wire. LV lead was positioned in Coronary Sinus, but while trying to slit the Coronary Sinus delivery catheter, LV lead dislodged.

**Fig. 2:**
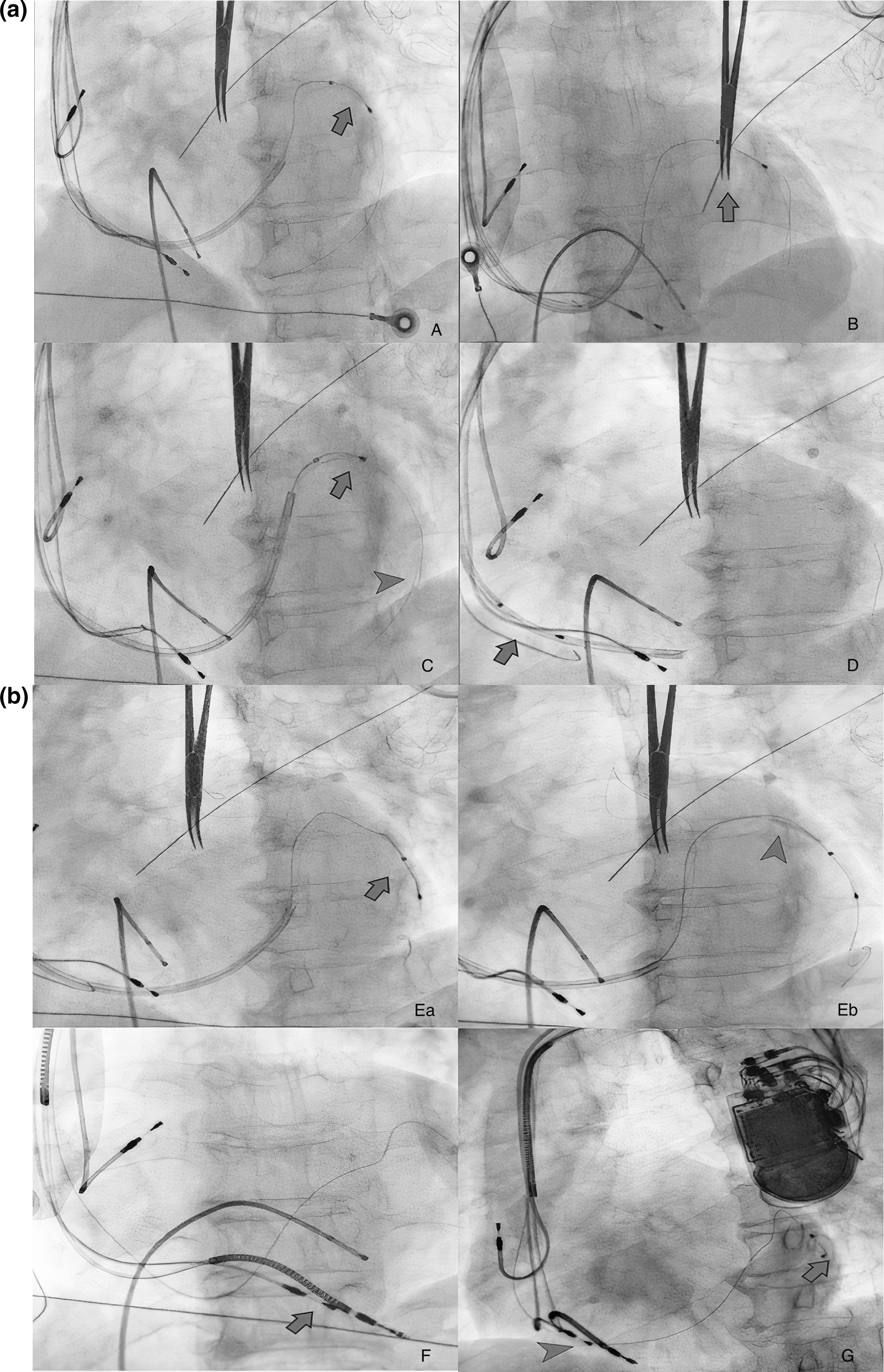
**a** (A) LV lead in Coronary Sinus (arrow), (B) While trying to slit the Coronary Sinus delivery catheter, LV lead dislodged (arrow), (C) Double wire (arrowhead) into the posterolateral vein, re-attempting to insert the LV lead (arrow) but LV lead could not be pushed more distally hence 1.5 × 8 mm balloon was used as support on second wire and LV lead could be pushed, (D) While trying to slit off the delivery catheter, LV lead (arrow) got dislodged during the second time again. **b** (Ea, Eb) LV lead (arrow) positioned more distally, and 2 × 12 mm balloon (arrowhead) inflated just distal to ostium of posterolateral vein, (F) Second ICD RV lead (arrow) was positioned and delivery sheath slit successfully, (G LAO view showing the position of all the leads. Note good separation in RV (arrowhead) and LV leads (arrow)

Two wires were again placed in the posterolateral vein. This time on one of the wires, a 1.5 × 8 mm balloon was passed distally and inflated to entrap the other wire. Following this, the LV lead could be advanced distally. The balloon was then deflated, and slitting of the delivery catheter was attempted, but again, the lead got dislodged. (Fig. 2A) On the third attempt again, two wires were placed in the posterolateral vein. An inflated balloon was used on one of the wires to trap the second wire and push the LV lead distally into the posterolateral vein. Subsequently, this balloon was deflated, removed and another balloon 2 × 12 mm was passed on the same wire. This balloon was placed proximal to the lead and inflated to entrap the LV lead so that it would not dislodge while slitting the delivery catheter. (Fig. 2B) After inflating the balloon, the sub-selector catheter was removed, and then the delivery catheter was slit. Then the balloon on the second wire was deflated and removed along with the second wire. With an anchor balloon inflated proximal to LV lead, the delivery sheath was slit successfully without lead dislodgement. The proximal anchor balloon effect enabled LV lead positioning at the midportion of the posterolateral vein. The leads were connected to a CRT pacemaker (Additional File 1). After the procedure, narrowing of the (110 ms) on ECG was observed (Fig. 1 post).

The patient underwent the procedure well without any complications and was discharged from the hospital the following day. The fluoroscopy time was 45 min. Device programming and lead values at the time of discharge from the hospital are summarized in Table 2.

**Table 2:** Device programming and lead values at the time of discharge from the hospital

Pacing parameter	Lead value	Atrium	RV	LV
DDD 50–130/min	Signal amplitude	3.0 mV	12 mV	17.1
SAV 100 ms	Pacing threshold	0.5 V	0.4 V	0.9 V
PAV 140 ms	Pacing impedance	540 Ω	660 Ω	535 Ω
LV → RV 30 ms				
Impulse amplitude		1.5 V (Auto)	2.0 V (Auto)	2.0 V (Auto)
Impulse width		0.5 ms	0.5 ms	0.5 ms
Sensitivity		0.3 mV (Auto)	0.5 mV (Auto)	0.6 mV

LV → RV, Interval between left ventricular and right ventricular pacing; PAV, paced AV interval; SAV, sensed AV interval

## Discussion and Conclusions

Although the patient had normal EF at the time of pacemaker implantation, he developed heart failure with reduced ejection fraction in a short timeframe. This fall in ejection fraction was due to right ventricular pacing (RVP), resulting in a lack of synchrony of the left ventricle due to mechanical and iatrogenic causes. RVP induces LV dysfunction and heart failure in around 10% to 20% of pacemaker patients, called pacing-induced cardiomyopathy [2, 3]. In such patients upgrading the device to CRT is a good option. Previously one study has reported that CRT upgrading significantly improves pacing-induced cardiomyopathy [4] while ongoing prospective trials like BUDAPEST-CRT Upgrade Study will provide crucial information on the role of CRT-D upgrade in such patients [5]. CRT is known to improve both symptoms and quality of life of the patient. Additionally, it also improves functional capacity and reverses LV remodeling. This results in the reduction of morbidity and mortality [6].

Despite advancement of technology and improvements in LV pacing leads and delivery systems, their placement goes on to be demanding due to complex coronary venous anatomy [7]. LV lead implantation failure occurs in 5–15% of the patients during attempted CRT implantation [8]. Primary reasons for the failure include inadequacy to cannulize the CS ostium, inadequacy to position the LV lead into a CS branch, phrenic nerve stimulation, or unsatisfactory pacing parameters. Balloons can enable the implantation of left ventricular (LV) lead, broadening the obstructions in the coronary sinus (CS) and small and/or stenotic coronary veins [9]. Similar procedure was described in the past with the distal anchor balloon technique [10]. This article describes an interesting case with complex coronary sinus anatomy and LV lead placement during CRT implantation due to tortuosity of the posterolateral vein. After multiple attempts, the proper lead placement was achieved with the use of an inflated balloon in the target vein as an anchor to enable initial coronary sinus cannulization and left ventricular lead placement. The interesting point, in this case, is that we inflated a balloon proximally to the lead to entrap it in the vein wall and thus slit the delivery sheath without lead dislodgement (proximal anchor balloon).

There are two ways to facilitate LV lead placement. One technique is to advance a vein selector over an angled Glidewire into the CS. Keeping the vein selector in the CS, the Glidewire is replaced with a long J-tip Extra Stiff wire that acts as a more robust rail to advance the CS sheath across the body of CS. Following this, smaller branches of CS are selectively cannulized to provide additional stability to the CS outer sheath during lead positioning. Besides conventional coronary vein interventions, the second technique available is to use the proximal anchor balloon, which was performed successfully in this case with angulated and tortuous coronary sinus branches, thus improving the success rate of LV lead placement for CRT. In this case, the perpendicular origin of the posterolateral vein and its proximity to the ostium of CS hampered the introduction of an LV delivery catheter and a wire. The guidewire did not provide adequate support for the placement of LV lead into the CS. With this approach, a small-caliber sheath was used to engage the ostiun of CS, through which an angioplasty wire was advanced into the body of CS. Then, a compliant coronary balloon was advanced over the angioplasty wire into the CS and into the candidate vessel for LV lead implantation, posterolateral vein. The balloon was then inflated to create an anchor. The procedure using balloons as anchor was initially described by Fujita et al. [11]. Another option is the ergonomic slitting technique which could enable the use of guide catheters for positioning endocardial leads by reducing the risk of lead dislodgement [12]. Additionally, an active fixation mechanism can be used to reduce the rate of lead dislodgement, but if its extraction is required, it can be problematic [13]. A transvenous approach for CRT implantation is routine practice. However, when transvenous coronary sinus lead placement or lead extraction is unsuccessful because of infection or lead failure, epicardial LV screw-in lead placement could be an option [14].

The proximal anchor balloon technique may improve LV lead placement success rate during CRT implantation in patients with difficult venous anatomy. This procedure is safe and can be easily applied. In conclusion, balloons can be used as anchors to facilitate CS cannulization and LV lead placement in patients with complex venous anatomy.

## Supplementary Information

**Additional file 1:** Full case recording.

## Data Availability

All data generated or analyzed during this study are included.

## Abbreviations

CRT: Cardiac resynchronization therapy
HF: Heart failure
CS: Coronary sinus
LV: Left ventricular
RV: Right ventricle
CRT-D: Cardiac resynchronization therapy defibrillator
AL-1: Amplatz left-1
WES: Whisper extra support
EF: Ejection fraction
RVP: Right ventricular pacing

## References

[1] Morgan JM, Delgado V (2009). Lead positioning for cardiac resynchronization therapy: techniques and priorities. Europace.

[2] Cho SW, Gwag HB, Hwang JK, Chun KJ, Park KM, On YK (2019). Clinical features, predictors, and long-term prognosis of pacing-induced cardiomyopathy. Eur J Heart Fail.

[3] Dor O, Haim M, Barrett O, Novack V, Konstantino Y (2020). Incidence and clinical outcomes of pacing induced cardiomyopathy in patients with normal left ventricular systolic function and atrioventricular block. Am J Cardiol.

[4] Barbieri F, Adukauskaite A, Heidbreder A, Brandauer E, Bergmann M, Stefani A (2021). Central sleep apnea and pacing-induced cardiomyopathy. Am J Cardiol.

[5] Merkely B, Kosztin A, Roka A, Geller L, Zima E, Kovacs A (2017). Rationale and design of the BUDAPEST-CRT Upgrade Study: a prospective, randomized, multicentre clinical trial. EP Europace.

[6] Sutton MS, Keane MG (2007). Reverse remodelling in heart failure with cardiac resynchronisation therapy. Heart.

[7] Pothineni NVK, Supple GE (2020). Navigating challenging left ventricular lead placements for cardiac resynchronization therapy. J Innov Card Rhythm Manag..

[8] Daoud EG, Kalbfleisch SJ, Hummel JD, Weiss R, Augustini RS, Duff SB (2002). Implantation techniques and chronic lead parameters of biventricular pacing dual-chamber defibrillators. J Cardiovasc Electrophysiol.

[9] Worley SJ (2009). How to use balloons as anchors to facilitate cannulation of the coronary sinus left ventricular lead placement and to regain lost coronary sinus or target vein access. Heart Rhythm.

[10] Kumagai Y, Arimoto T, Yamauchi S, Kutsuzawa D, Tsuchiya H, Watanabe M (2018). Implantation of a cardiac resynchronization therapy device using the anchor balloon technique in a patient with a tortuous coronary sinus branch. HeartRhythm Case Rep.

[11] Fujita S, Tamai H, Kyo E (2003). New technique for superior guiding catheter support during advancement of a balloon in coronary angioplasty: the anchor technique. Catheter Cardiovasc Interv.

[12] Lau EW (2008). An ergonomic guide catheter slitting technique designed to avoid lead dislodgement. J Interv Card Electrophysiol.

[13] Cronin EM, Ingelmo CP, Rickard J, Wazni OM, Martin DO, Wilkoff B (2013). Active fixation mechanism complicates coronary sinus lead extraction and limits subsequent reimplantation targets. J Interv Card Electrophysiol.

[14] Caliskan E, Fischer F, Schoenrath F, Emmert MY, Maisano F, Falk V (2017). Epicardial left ventricular leads via minimally invasive technique: a role of steroid eluting leads. J Cardiothorac Surg.

